# GSRF-DTI: a framework for drug-target interaction prediction based on a drug-target pair network and representation learning on a large graph

**DOI:** 10.1186/s12915-024-01949-3

**Published:** 2024-07-18

**Authors:** Yongdi Zhu, Chunhui Ning, Naiqian Zhang, Mingyi Wang, Yusen Zhang

**Affiliations:** 1 School of Mathematics and Statistics, Shandong University, Weihai, Shandong China; 2 Department of Central Lab, Weihai Municipal Hospital, Weihai, Shandong China

**Keywords:** Drug-target interaction, Graph representation learning, Graph neural network, Machine learning

## Abstract

**Background:**

Identification of potential drug-target interactions (DTIs) with high accuracy is a key step in drug discovery and repositioning, especially concerning specific drug targets. Traditional experimental methods for identifying the DTIs are arduous, time-intensive, and financially burdensome. In addition, robust computational methods have been developed for predicting the DTIs and are widely applied in drug discovery research. However, advancing more precise algorithms for predicting DTIs is essential to meet the stringent standards demanded by drug discovery.

**Results:**

We proposed a novel method called GSRF-DTI, which integrates networks with a deep learning algorithm to identify DTIs. Firstly, GSRF-DTI learned the embedding representation of drugs and targets by integrating multiple drug association information and target association information, respectively. Then, GSRF-DTI considered the influence of drug-target pair (DTP) association on DTI prediction to construct a drug-target pair network (DTP-NET). Next, we utilized GraphSAGE on DTP-NET to learn the potential features of the network and applied random forest (RF) to predict the DTIs. Furthermore, we conducted ablation experiments to validate the necessity of integrating different types of network features for identifying DTIs. It is worth noting that GSRF-DTI proposed three novel DTIs.

**Conclusions:**

GSRF-DTI not only considered the influence of the interaction relationship between drug and target but also considered the impact of DTP association relationship on DTI prediction. We initially use GraphSAGE to aggregate the neighbor information of nodes for better identification. Experimental analysis on Luo’s dataset and the newly constructed dataset revealed that the GSRF-DTI framework outperformed several state-of-the-art methods significantly.

**Supplementary Information:**

The online version contains supplementary material available at 10.1186/s12915-024-01949-3.

## Background

Identification of drug-target interactions can greatly improve the efficiency of drug discovery and development and reduce temporal and financial costs. Initially, researchers detected drug-target interactions using biological experiments, which have achieved good results, but large problems remain, including their adaptation to high throughput, low precision, and high cost [1]. Therefore, large-scale experiments cannot be widely used in practical applications to identify drug-target interactions. Fortunately, the development of information science and technology has promoted the flourishing of intelligent information processing technology such as machine learning, data mining, and mathematical statistics. Pushed by these technologies, computational approaches have established what is currently considered as the most effective method for drug-target interaction prediction [2, 3].

The existing computational-based methods of drug-target interaction identification can be divided into two categories: molecular docking-based methods [4] and machine-learning-based methods [5]. According to the principles of geometry complementarity and energy complementarity, molecular docking-based methods can effectively identify drug-target binding sites. However, these methods rely heavily on the three-dimensional structures of targets and are time-consuming [6]. Resting on the assumption of similarity, to wit, that similar drugs may interact with similar targets and vice versa [7], machine-learning-based methods are the most extensively used methods at the moment [8]. In recent decades, algorithms for DTI prediction have emerged one after another and fall into three main types: feature-based methods, network-based methods, and deep learning-based methods.

Feature-based DTI prediction models are classical methods that involve representing each drug/target as a vector of a certain length using biometric features. These features typically include the chemical structures and types of drugs, as well as the chemical and physical properties of targets, including their molecular structures. Based on the extracted feature vector, machine learning algorithms are used for the downstream prediction task. A bipartite local model (BLM), proposed by Bleakley and Yamanishi [9], is an approach based on a support vector machine, turning the DTI prediction problem into a binary classification problem. Then, Mei et al. [10] came up with a new model designed BLMNII by combining BLM with a neighbor-based interaction profile inferring procedure. BLM and BLMNII rely on drug-drug and target-target similarity. Xia et al. [11] introduced a semi-supervised learning model for DTI identification called NetLapRLS, which utilizes manifold regularization of labeled and unlabeled information by integrating known drug-protein interaction information, chemical structures of drugs, and genome sequence data. However, these methods do not take into consideration the interactions of drug-drug and target-target.

Network-based methods applied graph theory, which can clearly describe the interaction between different types of biological entities, thus making up for the shortcomings brought by the feature-based methods. Nascimento et al. [12] constructed a bipartite graph, in which drugs and targets are the nodes and the known DTIs are the edges, translating the DTI interaction problem into a link prediction task. Olayan et al. [13] extracted features based on a heterogeneous graph that contained known DTIs, drug-drug similarity and target-target similarity, and then applied an RF model to infer DTIs, developing a novel method designated DDR. Luo et al. [14] proposed an influential method called DTINet, which employed drugs, targets, diseases, side effects, and the association between two of them to construct a heterogeneous network in order to strengthen DTI prediction. DTINet focused on learning a low-dimensional feature representations of drugs and targets and makes predictions based on the representations via a vector space projection. However, these methods do not take into consideration the interaction of drug-target pairs.

Deep learning-based methods cleverly considered the association information between drug-target pairs and effectively identified DTIs. Deep learning algorithms, such as graph convolutional networks, graph attention networks, and autoencoder networks, have been effectively applied to construct DTI prediction models. Zhao et al. [15] learned the feature representation of each drug-protein pair (DPP); a heterogeneous network is built based on multiple drugs and targets, using a graph convolutional network. Then, they used a deep neural network to complete the DTI prediction. Li et al. [16] proposed a DTI prediction model DTI-MGNN based on multi-channel graph convolutional network and graph attention network. DTI-MGNN learned the semantic and topological features of DPP from the topology and feature graphs by using two independent graph attention networks and learned the common information of the two graphs using graph convolutional network (GCN).

It is difficult to fully capture network information or explore potential drug-target associations using only a single model invocation. Therefore, designing new hybrid methods is an effective way to study DTI prediction. In recent years, hybrid methods based on network and deep learning have made good progress in DTI prediction. On the basis of the constructed biological network, GCN uses convolution to fuse the structural features of graphs from a new perspective, which can capture the global information of the network to represent the features of nodes and achieve a more accurate prediction [17]. Nevertheless, the edge weight of GCN is fixed during fusion, which is not flexible enough. In addition, the scalability of GCN is poor, because of full-graph convolution fusion and full-graph gradient update, so when the graph is relatively large, there is an element of time complexity [18]. Compared with GCN, GAT [19] performs node feature fusion with an attention coefficient by adding a model learnable coefficient to each edge so that the model parameters can be adjusted according to the task in the process of convolutional feature fusion and become self-adaptive to achieve better results. However, GAT only integrates the information of first-order neighbors and does not go further into the information of higher-order neighbors [20]. In order to make up for the above deficiencies, GraphSAGE (Graph Sample and Aggregate) [21], a space-based algorithm, optimizes the sampling of the whole graph to the sampling of the current neighbor nodes, making the distributed training of large-scale graph data possible. GraphSAGE is also an inductive learning model that can directly calculate the invisible data in the training process without relearning the whole graph.

Here, we proposed a novel DTI prediction method called GSRF-DTI that integrated multiple types of networks and used GraphSAGE to learn feature representations of nodes. For details, we integrated drug/target-related networks to construct drug/target homogenous network. Considering the impact of the association relationships among DTPs on DTI prediction, we constructed DTP-NET. Therefore, the DTI prediction problem was transformed into the DTP classification problem, that is, the nodes classification task in DTP-NET. Here are three major contributions:

- GSRF-DTI considered not only the association between multiple biological entities but also the association between DTPs
- GSRF-DTI cleverly utilized GraphSAGE to make DTI prediction on large-scale graphs possible
- GSRF-DTI identified three novel DTIs. And the evaluation results indicate that the GSRF-DTI prediction method outperforms some state-of-the-art DTI prediction methods

## Methods

### Overview

**Fig. 1:**
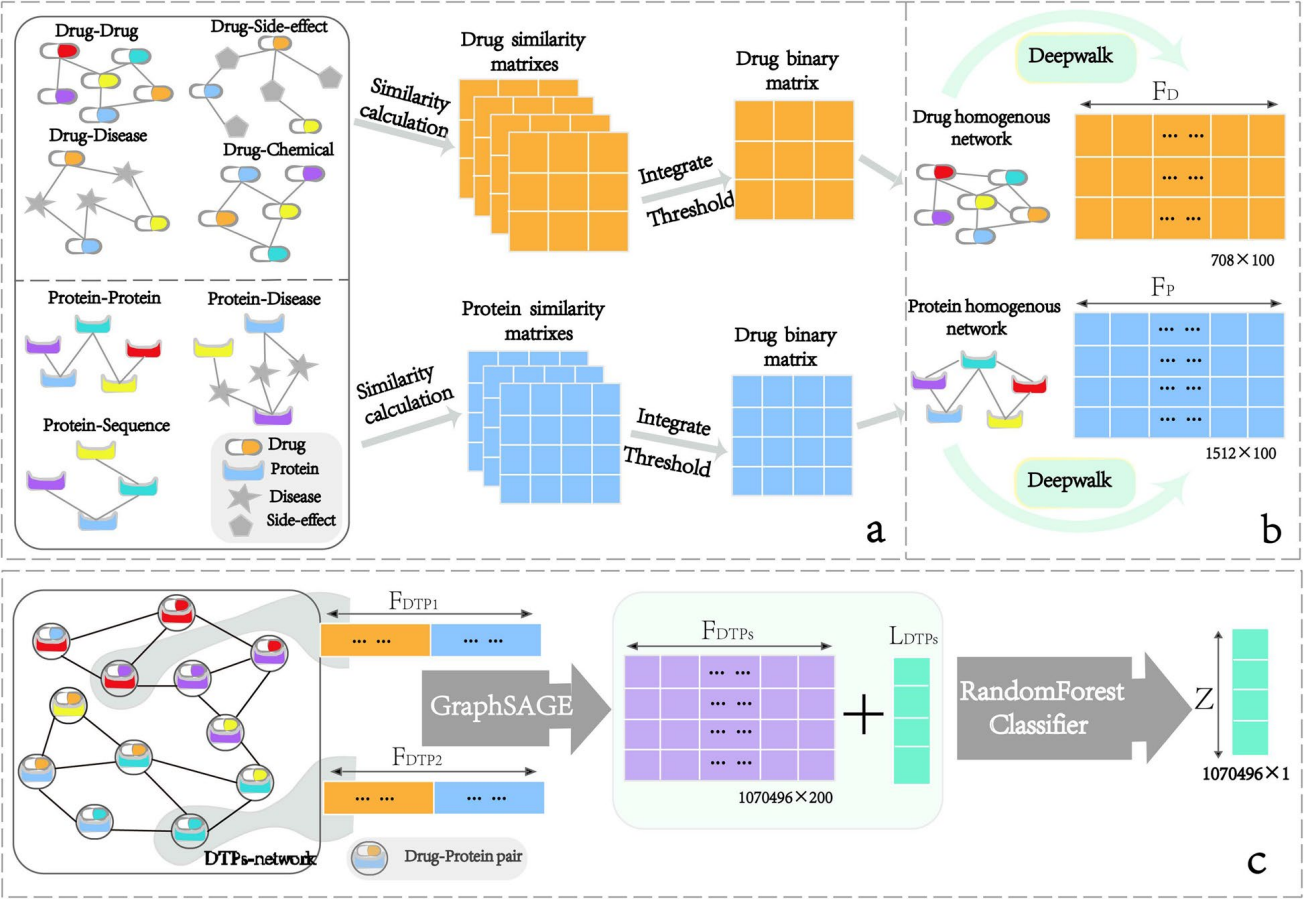
The workflow of GSRF-DTI. **a** Drug/target similarity matrices were constructed based on the similarity calculation of drug/ target-related networks, and then the similarity matrices were integrated into a drug/target homogeneous matrix. **b** The Deepwalk algorithm was applied to the homogeneous matrices to generate features ${F}_{D}$ and ${F}_{P}$ embedding of drugs and targets. **c** Construction of the drug-target pair network (DTP-NET). The initial features ${F}_{DTPs}$ of the network node consisted of the corresponding ${F}_{D}$ and ${F}_{P}$ directly concatenated, and the label ${L}_{DTPs}$ of the node was determined according to the known drug-protein interaction network. Then, GSRF-DTI used the GraphSAGE algorithm to obtain the new feature representation of drug-target pair nodes ${F}_{DTPs}{\prime}$. Finally, the random forest was used as a classifier to predict drug-target interactions (DTIs)

We proposed a novel hybrid method designated GSRF-DTI to identify DTIs. An overview of the GSRF-DTI model is shown in Fig. 1. GSRF-DTI mainly contains the following three parts. First, we constructed a drug homogeneous network and a target homogeneous network by integrating drug-related information and target-related information (Fig. 1a). Then, Deepwalk [22] was applied to the homogeneous networks established above for obtaining the topological features of drugs and targets (Fig. 1b). In the third step, GSRF-DTI constructed a DTP network (DTP-NET), in which a node represents a DTP and the edge represents the association between two DTPs; otherwise, the two DTPs will not be associated. The features representations of drugs and targets obtained in the second step were concatenated to form the initial node features (DTPs) in the DTP-NET. Finally, we applied the GraphSAGE algorithm on DTP-NET to update node features and train a classification model to achieve DTI prediction (Fig. 1c).

### Drug and target homogeneous network construction

Taking into account key factors in DTI prediction, such as biological entities like diseases, drug side effects, and drug-target interactions [23], we integrated seven types of network information.

According to previous research [14], both the Jaccard similarity coefficient and the Tanimoto coefficient are used to measure the similarity of two sets, but in cheminformatics, the Tanimoto coefficient is commonly used to assess the similarity between chemical structures. Additionally, when comparing the similarity of two sequences (DNA or protein sequences), the Smith-Waterman score is typically used. Therefore, we used the Jaccard similarity coefficient, Tanimoto coefficient, and Smith-Waterman score to evaluate the similarity between drugs/targets.

For drugs, three drug similarity matrices ${S}_{1}, {S}_{2}, {S}_{3}$ were obtained by calculating Jaccard similarity coefficient based on the drug-drug interaction network, drug-side effect association network, and drug-disease association network. The fourth drug similarity matrix ${S}_{4}$ was obtained by calculating the Tanimoto coefficient [24] based on the chemical structure of the drug. In the drug similarity matrices, we set a threshold $\alpha$ to construct the four drug binary matrices ${B}_{1}, {B}_{2}, {B}_{3}, {B}_{4}$. When $S_{ij}>\alpha$, we considered the two drugs to be similar, denoted ${B}_{ij}=1$; otherwise, ${B}_{ij}=0$. Based on the principle of “see one, get one,” the four binary matrices were integrated into the homogeneous network ${H}_{D}$.

**Fig. 2:**
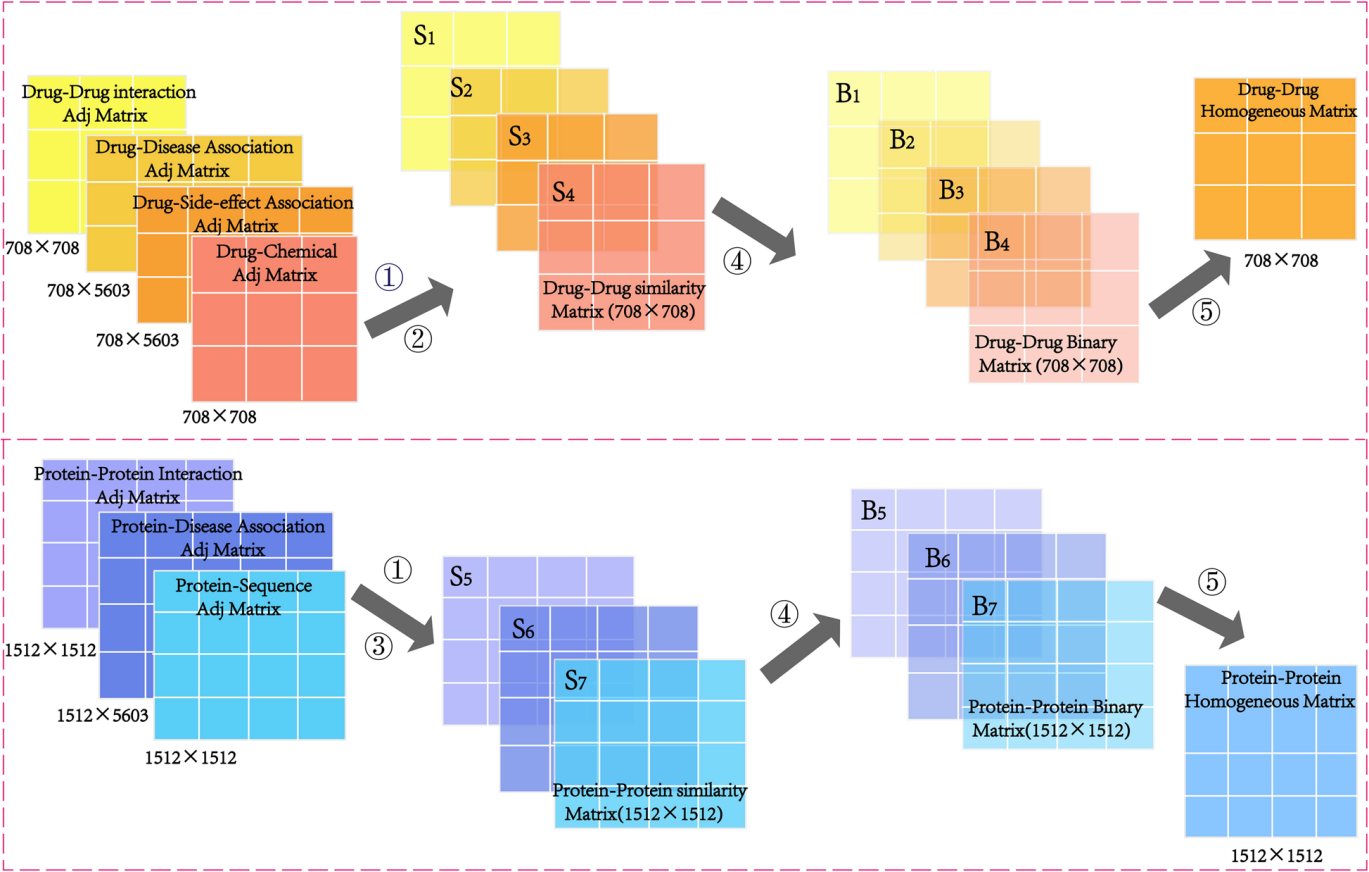
The construction process of the drug and target homogeneous network. (1) The drug similarity matrices ${S}_{1},{S}_{2},{S}_{3}$ and target similarity matrixes ${S}_{5},{S}_{6}$ were calculated by the Jaccard coefficient. (2) The drug similarity matrix ${S}_{4}$ was calculated by the Tanimoto coefficient. (3) The target similarity matrix ${S}_{7}$ was calculated by the Smith-Waterman score. (4) A suitable threshold $\alpha$ was set to construct binary matrices ${B}_{i},i=\text{1,2},\cdots ,7$. (5) The binary matrices were merged into homogeneous matrices ${H}_{D},{H}_{T}$ according to the principle of “see one, get one”

For targets, two target similarity matrices ${S}_{5},{S}_{6}$ were obtained by calculating Jaccard similarity coefficient based on the protein–protein interaction network and protein-disease association network. The third target similarity matrix ${S}_{7}$ was obtained by calculating the Smith-Waterman score [25] based on the sequence information of the protein. In the same way as we constructed the drug binary matrix, we obtained three target binary matrices ${B}_{5},{B}_{6},{B}_{7}$, to construct the homogeneous network ${H}_{T}$. The above process is shown in Fig. 2.

### Deepwalk-based representation learning

Deepwalk is a graph embedding algorithm whose function is similar to Word2vec [26]. It uses the co-occurrence relationship between nodes in the graph to learn the vector representation of nodes.

Deepwalk mainly includes two parts: random walk and generating embedding representation. In the homogeneous networks ${H}_{D}$ and ${H}_{T}$, Deepwalk performed random walk [27] sampling from each node to obtain locally associated training data. Subsequently, SkipGram [28] training was performed on the sampled data, and then the discrete network nodes were vectorized to obtain the drug feature representation ${F}_{D}$ and the target feature representation ${F}_{T}$.

The graph embedding algorithm achieves a low-dimensional representation of nodes in the network. In this way, it effectively preserves the topology and node information of the network and reduces the information loss of nodes.

### DTP network construction

Considering the influence of the association between DTPs on DTI prediction, we constructed a DTP-NET based on the drug set and target set. In DTP-NET, each DTP consisted of a drug and a target, representing a node in the network. Therefore, the number of nodes in the network is:

1 $${N}_{DTP}={N}_{D}\times {N}_{T}$$

where ${N}_{DTP}$ denotes the number of nodes in the DTP-NET, ${N}_{D}$ denotes the number of drugs, and ${N}_{T}$ is the number of targets.

For the edges of the DTP-NET, we defined an edge between two DTPs if they shared a common drug or target. Otherwise, no connection was established between them. Using ${D}_{i}{T}_{j}P$ to represent the interaction between the $i-th$ drug and $j-th$ target, the above definition is described as follows:

2 $$f({D}_{i}{T}_{j}P,{D}_{p}{T}_{q}P)\hspace{0.33em}=\hspace{0.33em}\left\{\begin{array}{c}1,\\ 0,\end{array}\right.\begin{array}{c} \, \hspace{0.33em}\hspace{0.33em}\hspace{0.33em}i=p\hspace{0.33em}{\text{or}}\hspace{0.33em}j=q\\ {\text{otherwise}}\end{array}$$

Thus, the adjacency matrix $A$ of the DTP-NET can be expressed as:

3 $$A={\left[\begin{array}{cccc}f({D}_{1}{T}_{1}P,{D}_{1}{T}_{1}P)& f({D}_{1}{T}_{1}P,{D}_{1}{T}_{2}P)& ...& f({D}_{1}{T}_{1}P,{D}_{{N}_{D}}{T}_{{N}_{T}}P)\\ f({D}_{1}{T}_{2}P,{D}_{1}{T}_{1}P)& f({D}_{1}{T}_{2}P,{D}_{1}{T}_{2}P)& ...& f({D}_{1}{T}_{2}P,{D}_{{N}_{D}}{T}_{{N}_{T}}P)\\ ...& ...& ...& ...\\ f({D}_{{N}_{D}}{T}_{{N}_{T}}P,{D}_{1}{T}_{1}P)& f({D}_{{N}_{D}}{T}_{{N}_{T}}P,{D}_{1}{T}_{1}P)& ...& f({D}_{{N}_{D}}{T}_{{N}_{T}}P,{D}_{{N}_{D}}{T}_{{N}_{T}}P)\end{array}\right]}_{{N}_{DTP}\times {N}_{DTP}}$$

Based on the feature representation of the drug and target learned by Deepwalk, we obtained the feature representation of DTPs though feature concatenation. In detail, using ${D}_{i}{T}_{j}P$ as an example, its initial feature ${F}_{{D}_{i}{T}_{j}P}$ is represented by the following formula:

4 $${F}_{{D}_{i}{T}_{j}P}\hspace{0.33em}=\hspace{0.33em}{F}_{{D}_{i}}||{F}_{{T}_{j}}$$

where “$||$” is the concatenation of the drug feature ${F}_{{D}_{i}}$ and target feature ${F}_{{T}_{j}}$.

For the label ${L}_{{D}_{i}{T}_{j}P}$ of node ${D}_{i}{T}_{j}P$, if there is a known interaction between ${D}_{i}$ and ${T}_{j}$, ${L}_{{D}_{i}{T}_{j}P}=1$; otherwise, ${L}_{{D}_{i}{T}_{j}P}=0$, i.e.,

5 $${L}_{{D}_{i}{T}_{j}P}=\left\{\begin{array}{c}1,\\ 0,\end{array}\right.\hspace{0.33em}\hspace{0.33em}\hspace{0.33em}\begin{array}{c}\hspace{0.33em}\hspace{0.33em}{D}_{i}\hspace{0.33em}{\text{interact}} \hspace{0.33em}{\text{with}}\hspace{0.33em}{T}_{j}\hspace{0.33em}\hspace{0.33em}\hspace{0.33em}\hspace{0.33em}\hspace{0.33em}\hspace{0.33em}\hspace{0.33em}\hspace{0.33em}\\ \hspace{0.33em}\hspace{0.33em}{D}_{i}\hspace{0.33em}{\text{is}}\hspace{0.33em}{\text{not}}\hspace{0.33em}{\text{interact}} \hspace{0.33em}{\text{with}}\hspace{0.33em}{T}_{j}\end{array}$$

As a result, GSRF-DTI is a supervised learning framework to identify DTIs.

### GraphSAGE-based potential network feature learning

In this section, we introduce how to perform DTI prediction and model optimization. After constructing the DTP-NET and obtaining the initial features of DTPs, we used the GraphSAGE algorithm to learn the potential network features. The learned features were fed into a random forest classifier for DTI prediction.

Specifically, GraphSAGE, an inductive framework, efficiently produced potential network features by sampling and aggregating the features of local neighbor nodes. By extension, the K-layer neighbor node information of the central node ${D}_{i}{T}_{j}P$ was taken, which was aggregated to gain the feature of ${D}_{i}{T}_{j}P$. Assuming that we have learned the $k-th,\hspace{0.33em}\forall k\in \{\text{1,2},\cdots ,K\}$ aggregation function $AGGREGAT{E}_{k}$, as well as the weight matrix ${W}^{k}$, the features of ${D}_{i}{T}_{j}P$ neighbor nodes at the layer $k$ were obtained by the following Formula (6):

6 $${h}_{N({D}_{i}{T}_{j}P)}^{k}=AGGREGAT{E}_{k}(\{{h}_{{D}_{m}{T}_{n}P}^{k-1},\forall {D}_{m}{T}_{n}P\in {N}_{k}({D}_{i}{T}_{j}P)\})$$

where ${N}_{k}({D}_{i}{T}_{j}P)$ denotes the set of neighbor nodes in the $k\text{-th}$ layer of ${D}_{i}{T}_{j}P$, ${h}_{{D}_{m}{T}_{n}P}^{k-1}$ represents the feature of ${D}_{m}{T}_{n}P$ in the $k-1\text{-th}$ layer, and $AGGREGAT{E}_{k}(\cdot )$ represents the selected aggregation function. There are three commonly used aggregation functions, namely, the mean aggregator, pooling aggregator, and LSTM aggregator [21].

In feature integration theory, concatenating ${h}_{N({D}_{i}{T}_{j}P)}^{k}$ and ${h}_{{D}_{i}{T}_{j}P}^{k-1}$ that is the feature of ${D}_{i}{T}_{j}P$ at the $k-1\text{-th}$ layer and then through a nonlinear transformation $\sigma$ to get the ${D}_{i}{T}_{j}P$ feature ${h}_{{D}_{i}{T}_{j}P}^{k}$ at the $k\text{-th}$ layer, is shown in Formula (7):

7 $${h}_{{D}_{i}{T}_{j}P}^{k}=\sigma ({W}^{k}\cdot CONCAT({h}_{{D}_{i}{T}_{j}P}^{k-1},{h}_{N({D}_{i}{T}_{j}P)}^{k}))$$

To learn valid, predictive representations, we adopted the cross-entropy loss function in Formula (8) to train the model and output the feature ${h}_{{D}_{i}{T}_{j}P}^{k}$, the weight matrices ${W}^{k}$, and reference parameters of aggregate functions.

8 $$Loss=-\frac{1}{n}\sum\limits_{i}^{n}\left[{y}_{i}\log\widehat{y}_{i}+(1-y_{i})\log(1-\widehat{y}_{i})\right]$$

where ${y}_{i}$ represents the real value, and ${\widehat{y}}_{i}$ represents the predictive value.

**Fig. 3:**
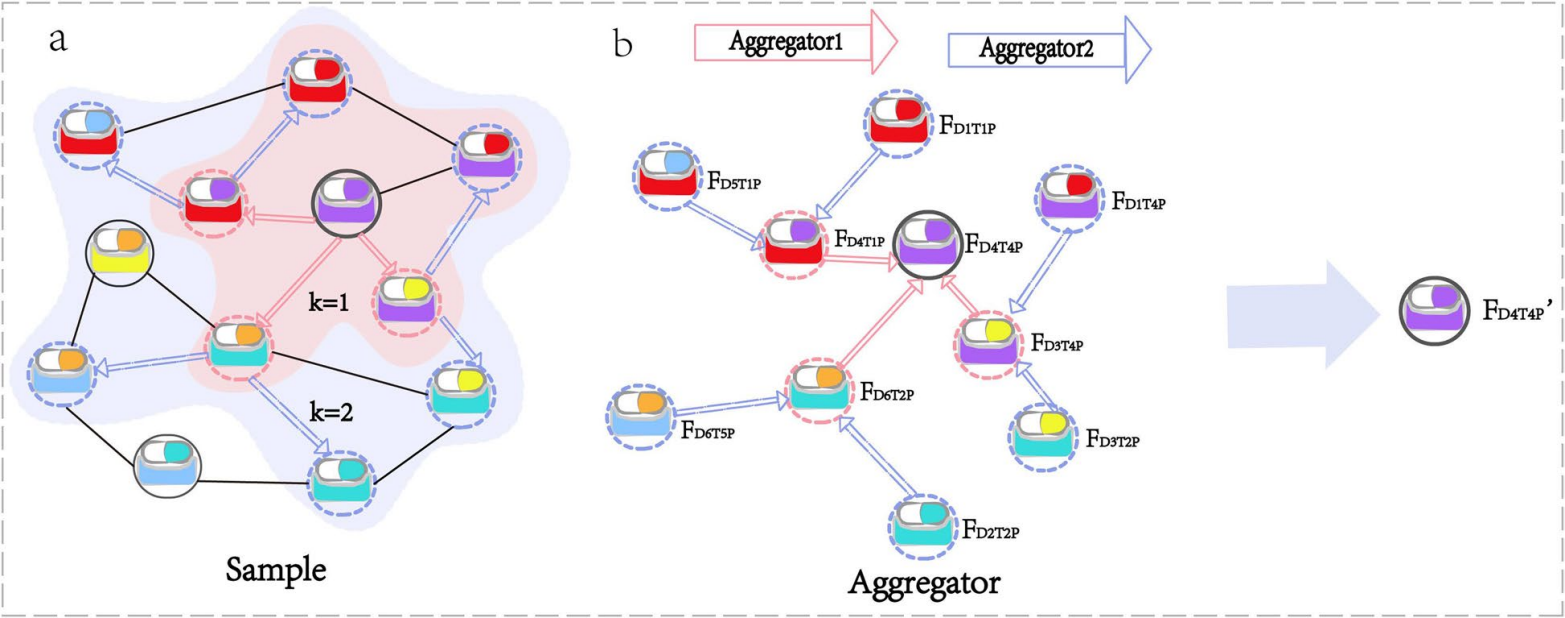
Visual illustration of GraphSAGE. **a** Sampling process. **b** Aggregation process. In **b**, ${F}_{{D}_{4}{T}_{4}P}$ represents the features of the node ${D}_{4}{T}_{4}P$, and ${F}_{{D}_{4}{T}_{4}P}{\prime}$ represents the features of ${D}_{4}{T}_{4}P$ through sampling and aggregation

William L Hamilton’s research showed that GraphSAGE could achieve high performance with $K=2$ and ${S}_{1}\cdot {S}_{2}\le 500$, where ${S}_{i}$ denotes the number of sampled $i\text{-th}$ layer neighbor nodes. Therefore, in our model GSRF-DTI model, we set *K* = 2, ${S}_{1}=50$, and ${S}_{2}=10$. The framework diagram of GraphSAGE is shown in Fig. 3.

### Classification by random forest

The construction of DTP-NET transformed the problem of link prediction between nodes in heterogeneous graphs into the problem of node classification in DTP-NET.

In the previous sections, we acquired the potential network features and labels of DTPs. Then, we used random forest [29], which is one of the classical binary classification algorithms, to deal with the binary classification task and identify DTIs.

We trained a random forest classifier, where the input was the representation ${F}_{DTPs}{\prime}$ of DTPs, and the output is the probability of interaction between drug and target. The Gini coefficient was used as the training metric during the training process.

## Results

### Datasets

To evaluate the performance of a drug-target interaction prediction algorithm based on a drug-target bipartite network and graph representation learning, we tested our model on Luo et al. dataset [14] as well as on a newly constructed dataset. Detailed data for the above two datasets were provided in Additional file 1.

Luo’s dataset contains a total of four biological entities (drugs, proteins, diseases, and side effects). These entities constitute a total of seven interaction networks that GSRF-DTI uses to construct homogeneous networks of drugs and targets for subsequent DTI prediction.

Among them, drug entities were collected from the DrugBank database (Version 3.0) [30], protein entities were collected from the HPRD database (Release 9) [31], disease entities were collected from the Comparative Toxicogenomics database [32], and side effects entities were collected from the SIDER database (Version 2) [33]. A detailed description of the number of various biological entities and their interactions is provided in Additional file 2: S1.1. In addition, we used the chemical structure of the drugs and the similarity information of the protein sequence. The chemical structures of drugs were downloaded from the DrugBank database (Version 3.0), and the protein sequence was downloaded from the integrated medicinal genomic database of Sophic [34].

The newly constructed dataset similarly contains four types of entities (drugs, proteins, diseases, and side effects). Inspired by [14] and [9], the sources of drug-disease, drug-side effect, and protein-disease association information were the same as those of Luo’s dataset. Then, chemical structures of the drugs were obtained from the DRUG and COMPOUND Sections in the KEGG LIGAND database [35]. Amino acid sequences of the target proteins were obtained from the KEGG GENES database. Finally, known drug-protein interactions were obtained from KEGG BRITE, BRENDA [36], SuperTarget [37], and DrugBank databases; we focused only on regulatory interactions between enzymes and compounds.

Since different data sources use different identifiers for the same entity, we utilized BioKG [38] to parse drugs and proteins identities of different data sources into unified DrugBank ids and UniProt ids, respectively. Based on the unified IDs, we only retained drugs that have disease, side effect, and chemical structure information simultaneously as well as proteins that have disease and amino acid sequence information simultaneously. A detailed description of the number of various biological entities and their interactions is provided in Additional file 2: S1.2.

### Data generation

The evaluation dataset was generated in the same way as the method used in EEG-DTI [39]. In terms of expansion, for Luo’s dataset, this dataset contains 708 drugs and 1512 targets; the DTP-NET consisted of 1,070,496 $(708 \times 1512)$ nodes. We labeled the nodes corresponding to the 1923 known drug-target interactions as 1, considering them as positive samples. Next, we randomly selected nodes with an equal number of positive and negative samples from the remaining 1,068,573 nodes labeled as 0. For the newly constructed dataset, there are 151 drugs and 285 proteins, so the DTP-NET constructed based on this has a total of 43,035 $(151\times 285)$ nodes, of which 481 are known drug-target interactions.

To minimize the influence of data variability on the results, we used fivefold cross-validation to evaluate our model. Positive samples and negative samples were divided into 5 parts. Then, one positive part and one negative part were selected as the test sets every time, and the remaining parts were successively selected as the training set. Finally, the average value of the five results was calculated as the final evaluation metric.

### Performance evaluation on Luo dataset

To comprehensively evaluate the performance of GSRF-DTI, we used the area under the receiver operating characteristic curve (AUROC) and the area under the exact recall curve (AUPR) as evaluation index, similar to previous work [15, 17, 39]. We compared GSRF-DTI with five state-of-the-art DTI prediction approaches on Luo’s dataset, including EEG-DTI [39], GCN-DTI [15], BLMNII [10], NRLMF [40], and DTI-NET [14]. The introduction of approaches proposed above is provided in Additional file 2: S2.1. In addition, the specific parameter settings in GSRF-DTI were shown in Additional file 2: S2.2*.*

**Fig. 4:**
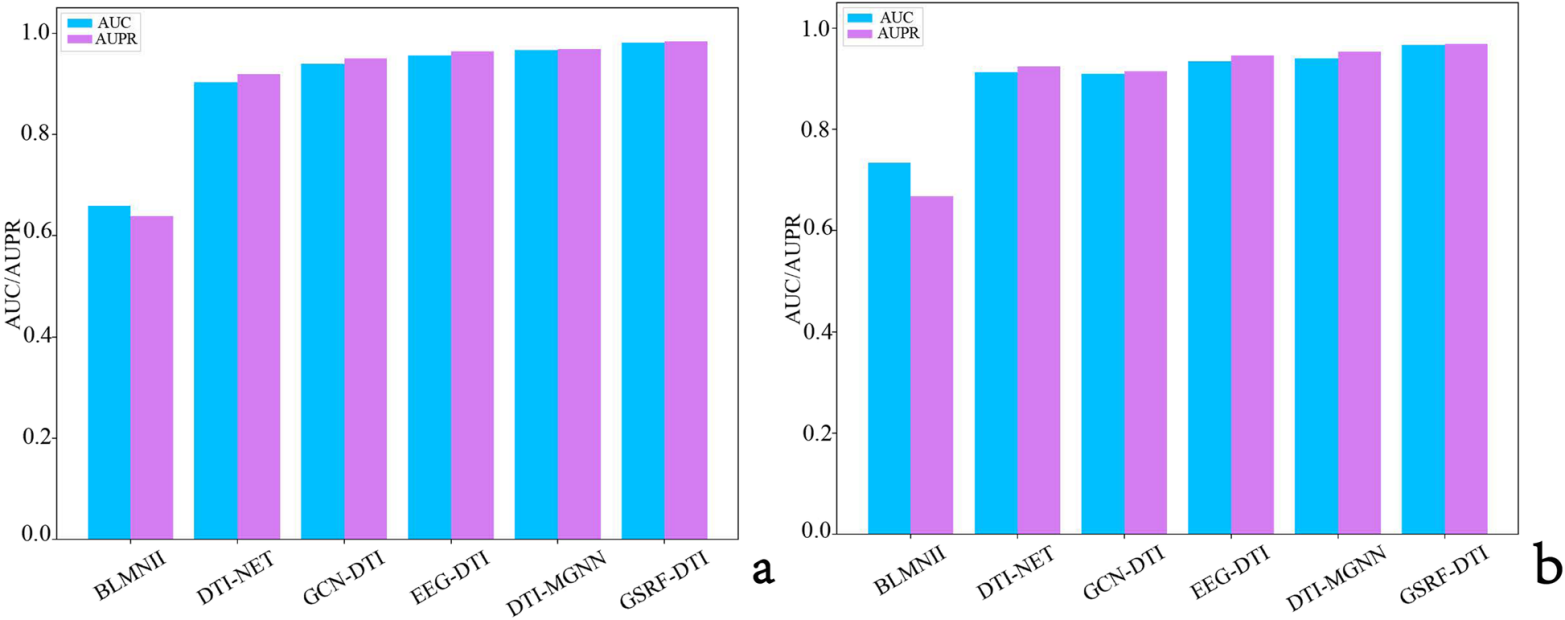
AUROC and AUPR performance of different methods. **a** AUROC and AUPR performance of different methods on Luo’s dataset. **b** AUROC and AUPR performance of different of different methods on the newly constructed dataset

The comparative results are shown in Table 1. GSRF-DTI consistently outperformed the other five baseline methods, with AUROC and AUPR values of up to 97.78% and 98.04%, respectively. These AUROC and AUPR values were 2.59% and 1.94% higher, respectively, compared to EEG-DTI, which ranked as the third-best approach. The potential reason is that GSRF-DTI additionally considers the association relationship between DTPs. Compared to GCN-DTI, GSRF-DTI achieved a 4.27% higher AUROC and a 3.32% higher AUPR. GSRF-DTI may have a greater consideration of the multiple interrelated interactions between biological entities in the process of drug and target feature representation learning. The visual representation of the results is shown in Fig. 4a.

**Table 1:** AUROC and AUPR results of DTI prediction from the different methods on Luo’s dataset

Method	AUROC	AUPR
BLMNII	0.6595 ± 7.8e − 3	0.6382 ± 3.6e − 4
DTI − NET	0.9030 ± 4.8e − 3	0.9187 ± 7.2e − 4
GCN-DTI	0.9391 ± 5.3e − 4	0.9507 ± 4.7e − 3
EEG-DTI	0.9559 ± 3.5e − 3	0.9645 ± 5.8e − 3
DTI-MGNN	0.9665 ± 6.2e − 4	0.9683 ± 4.1e − 4
**GSRF-DTI**	**0.9818 ± 4.8e − 3**	**0.9839 ± 5.1e − 3**

### Performance evaluation on the newly constructed dataset

For further evaluation, we constructed a new dataset, implemented GSRF-DTI algorithm, and compared it with other five methods. The experimental results are shown in Table 2.

**Table 2:** AUROC and AUPR values of DTI prediction from the different methods on the newly constructed dataset

Method	AUROC	AUPR
BLMNII	0.7341 ± 2.5e − 3	0.6678 ± 3.1e − 4
DTI − NET	0.9129 ± 4.8e − 3	0.9238 ± 7.2e − 4
GCN-DTI	0.9092 ± 5.3e − 4	0.9147 ± 4.7e − 4
EEG-DTI	0.9348 ± 3.5e − 4	0.9462 ± 5.8e − 3
DTI-MGNN	0.9402 ± 6.2e − 3	0.9533 ± 4.1e − 3
**GSRF-DTI**	**0.9666 ± 2.5e − 3**	**0.9685 ± 2.1e − 3**

Comparing the results in Table 1, it is evident that the performance of models based on deep learning, such as GSRF-DTI, DTI-MGNN, EEG-DTI, and GCN-DTI, in predicting DTI on the newly constructed dataset has slightly decreased. This can be attributed to the fact that deep learning algorithms generally perform better on large sample problems. The results in Table 2 show that the AUROC and AUPR values of GSRF-DTI reach as high as 96.66% and 96.85% respectively, both outperforming the other five baseline methods, thus further emphasizing the effectiveness of our proposed method GSRF-DTI in identifying DTIs. The visual representation of the results is shown in Fig. 3b.

### Sensitivity analysis

#### The effects of learning_rate

**Fig. 5:**
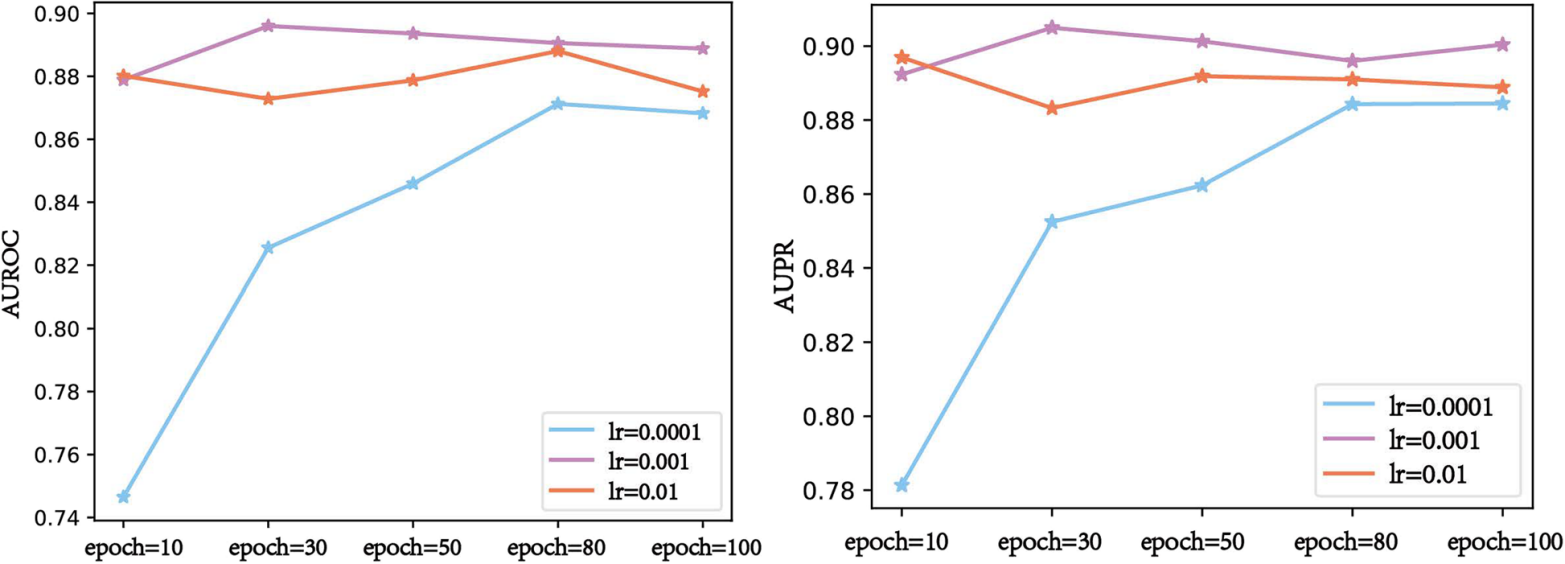
AUROC and AUPR values of the different learning rates

The learning rate is an important parameter in supervised learning and deep learning, which determines the step size of the weight change and affects the convergence of the objective function. To obtain the optimal model, we set different learning rates to train GSRF-DTI. The detailed results are shown in Additional file 2: S3.1, and the average AUROC and AUPR values of fivefold cross-validation are shown in Fig. 5.

From Fig. 5, we can conclude that AUROC and AUPR values of the model prediction results were the highest when the learning rate was 0.001. We continuously compared the effects of different values of learning rate on the model performance and finally set learning rate to 0.001.

#### The effects of the aggregate function

**Fig. 6:**
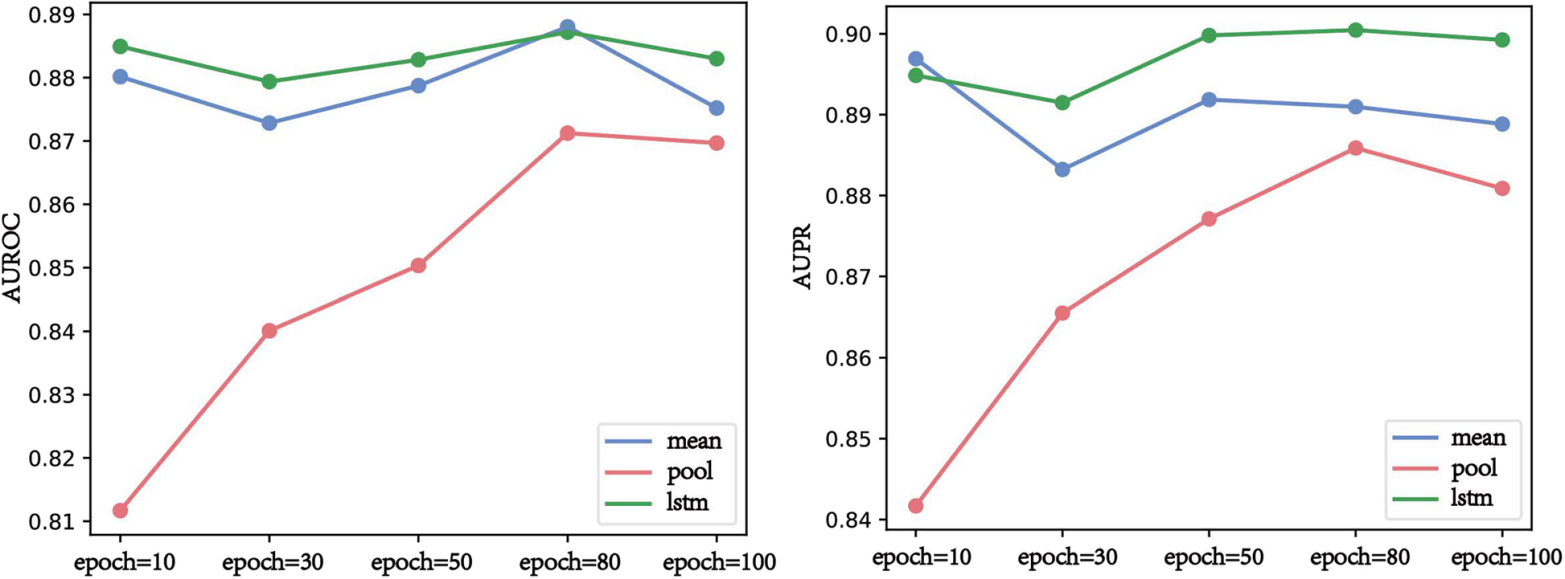
AUROC and AUPR values from the different aggregators

The aggregation function directly affects the feature representation of nodes in the DTP-NET, which indirectly affects DTI prediction. The GraphSAGE algorithm usually has three commonly used aggregation functions: the mean aggregator, pooling aggregate, and LSTM aggregator. To assess the impact of different aggregation functions on model performance, we calculated the evaluation index of the model prediction results under each aggregation function. The detailed results are shown in Additional file 2: S3.2, and the average AUROC and AUPR values of fivefold cross-validation are shown in Fig. 6. Note that we set learning_rate to 0.001 at this point.

Figure 6 shows that the performance of the LSTM aggregator was slightly better. However, the time complexity of the LSTM aggregator was much larger than that of the other two aggregators. Considering the time complexity and model performance, we chose the mean aggregator as the aggregation function of our GSRF-DTI model.

#### The effects of the classifier

The classical algorithms in machine learning are logistic regression (LR) [41], support vector machine (SVM) [42], and random forest (RF) [29], which are commonly used to work out binary classification tasks. To obtain better model performance, we took the node label ${L}_{DTPs}$ and the node features ${F}_{DTPs}{\prime}$ sampled and aggregated by the GraphSAGE algorithm as the input of the above classification algorithms to train the model and predict the DTIs. To avoid the occasionality of the results, we randomly divide the dataset 50 times, namely, 75% as the training set and 25% as the validation set. The average of the results was used as the final index value, as shown in Table 3.

**Table 3:** AUROC and AUPR values from the different classification algorithms

Classification methods	LR	SVM	RF
AUROC	0.9798	0.9800	0.9818
AUPR	0.9808	0.9809	0.9839

The experimental data showed that RF performed the best in classification; therefore, RF was determined as the classification method of our model for DTI identification.

### Ablation experiment

#### The effects of different types of network information

One of the innovative points of the GSRF-DTI proposed in this paper is the integration of seven types of network information. In order to evaluate the importance of each type of network on DTI prediction, we designed five ablation experiments on Luo’s dataset. The experimental findings are summarized in Table 4.

**Table 4:** Evaluation metric values of the ablation experiments

Setting	AUROC	AUPR
ALL networks (GSRF-DTI)	**0.9818**	**0.9839**
Without side effects	0.9816	0.9838
Without chemical structures of drugs	0.9797	0.9808
Without diseases	0.9781	0.9830
Without diseases and side effects	0.9776	0.9817
Without sequence information of proteins	0.9757	0.9784

From Table 4, it is evident that the model performed best when utilizing all networks. When protein sequence information was removed, the model exhibited the poorest performance in predicting DTI. This suggests that among the seven types of information considered, protein sequence information was the most important for accurately identifying DTI. When removing drug chemical structures or side effects, the AUROC and AUPR decreased, but only slightly. However, when removing diseases, AUROC and AUPR experienced a significant decrease. This could be attributed to diseases being associated with both drugs and proteins, whereas chemical structures and side effects are exclusively related to drugs. Finally, if side effects and diseases were removed simultaneously, the AUROC and AUPR decreased compared to when diseases or side effects were removed individually.

All the ablation experiments conducted above provide evidence that the integration of the seven types of information can enhance the identification performance of DTI.

#### Evaluation of the effectiveness of the GraphSAGE algorithm

We propose that the GSRF-DTI model is suitable for feature representation learning on large-scale networks. To further illustrate the effectiveness of the GraphSAGE algorithm, we designed three comparative experiments. Specifically, in the basic experiment, we identified DTIs limited to drug and target-related information, that is, the initial features ${F}_{DTPs}$ of DTPs obtained by concatenating the corresponding drug feature ${F}_{D}$ and target feature ${F}_{T}$ after the action of the Deepwalk were directly used as the input of the three classification algorithms. In the contrast experiment, the association between DTPs was further considered, that is, the DTP features ${F}_{DTPs}{\prime}$ after the GraphSAGE that was applied to the DTP-NET were used as the input of the three classification algorithms. We evaluated the model performance by calculating the following six evaluation indicators: accuracy, precision, recall, F1 score, AUROC, and AUPR. The detailed results are shown in Additional file 2: S4, and the average values of fivefold cross-validation are shown in Table 5.

**Table 5:** The six evaluation metric values of the comparative experiments

Evaluation metrics	Methods					
	Deepwalk + LR	Deepwalk + GraphSAGE + LR	Deepwalk + SVM	Deepwalk + GraphSAGE + SVM	Deepwalk + RF	Deepwalk + GraphSAGE + RF
Accuracy	0.7750	0.9545	0.8475	**0.9581**	0.8577	0.9578
Precision	0.7853	0.9555	0.8843	**0.9634**	0.8947	0.9626
Recall	0.7533	**0.9542**	0.7975	0.9532	0.8089	0.9533
F1 Score	0.7687	0.9548	0.8384	**0.9582**	0.8494	0.9579
AUROC	0.8494	0.9798	0.9123	0.9800	0.9213	**0.9818**
AUPR	0.8521	0.9808	0.9214	0.9809	0.9308	**0.9839**

**Fig. 7:**
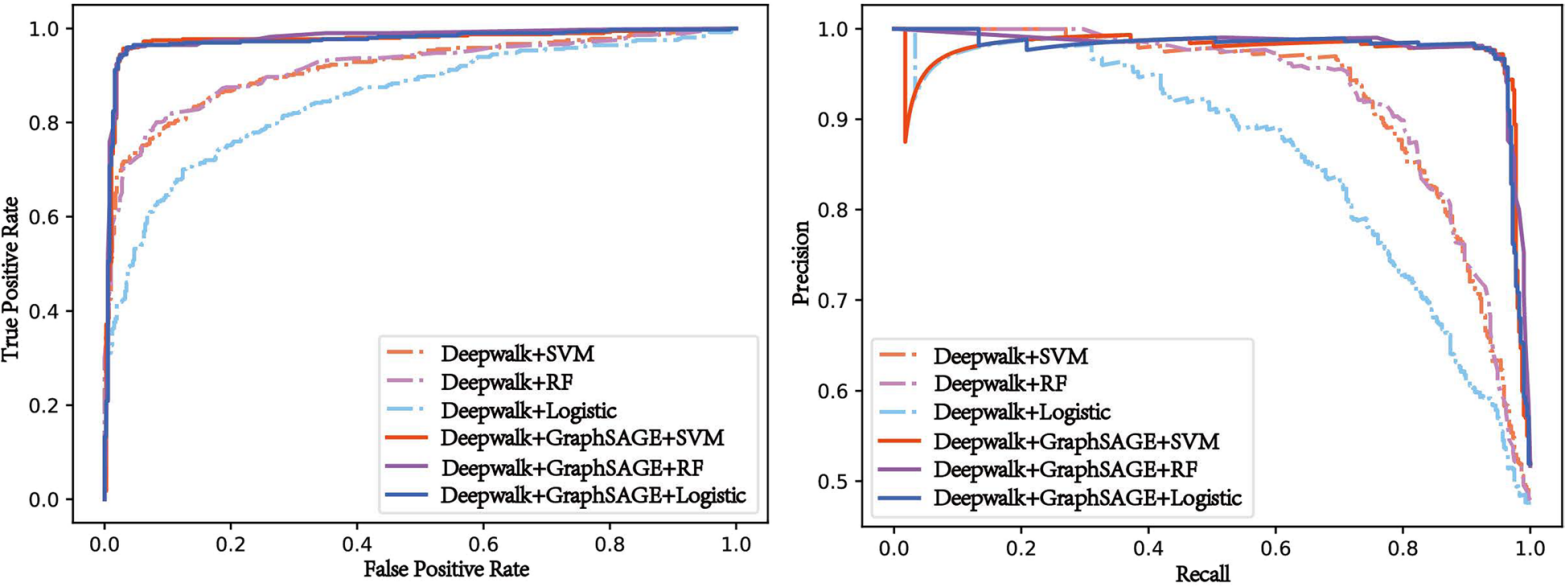
ROC curves and PR curves of the comparative experiments

Table 5 depicts that further considering the association information between DTPs was far better than the experimental results that only considered the drug/target association information. The model prediction results of the three classical classification algorithms combined with GraphSAGE were higher than those of each classification algorithm alone. Thus, the effectiveness of the GraphSAGE algorithm was more strongly illustrated. In this paper, GraphSAGE combined with RF was finally applied to predict DTIs. Table 5 also depicts that the AUROC and AUPR values of the model (Deepwalk + GraphSAGE + RF) prediction were the highest. Specifically, GSRF-DTI achieved an AUROC score of 0.9818 and an AUPR score of 0.9839. To compare the results of the basic experiment and the contrast experiment more intuitively, ROC and PR curves were drawn and shown in Fig. 7.

Based on the results in Fig. 7, the area under all the solid lines exceeded that under the dotted lines. As previously mentioned, the classification algorithm combined with the GraphSAGE algorithm exhibited slightly superiority, indicating the effectiveness of GraphSAGE for inductive representation learning of large graphs. It also shows that the consideration of the interaction information between DTPs played an important role in DTI prediction.

## Case study

So far, we have demonstrated the effectiveness of the GSRF-DTI in predicting drug-target interactions. Finally, we used GSRF-DTI to predict drugs and proteins with potential interactions. By ranking drug-target pairs according to predicted scores, we identified the top 100 as potential DTIs identified by GSRF-DT, of which 69 were already known DTI and could be found in DrugBank. Through a literature search and analysis of the remaining 31 DTIs, we identified three novel DTIs:

(i) Kim et al. [43] showed that the administration of Triamcinolone significantly reduced the expression level of the leukotriene C4 synthase gene (LTC4S). This is a potential DTI identified by GSRF-DTI, but the interaction was not found in DrugBank
(ii) Kotridis et al. [44] indicated that Irbesartan exert part of their antihypertensive action by increasing atrial natriuretic peptide plasma (NPR1) levels. This is a potential DTI identified by GSRF-DTI
(iii) Turrell et al. [45] investigated the role of ATP-sensitive inward rectifier potassium channel 11 (KCNJ11) in the preconditioning of phenylephrine in isolated ventricular myocytes. The interaction between phenylephrine and KCNJ11 was also identified by GSRF-DTI without a positive result in DrugBank

These case studies show the reliability of the results obtained from GSRF-DTI and illustrate its ability to identify interactions between drugs and proteins.

## Discussion

Large-scale experimental approaches can test only one chemical at a time to identify interacting proteins, and they entail high costs. In contrast, computational methods can evaluate multiple promising drug candidates simultaneously, greatly improving the efficiency of drug target identification. Therefore, we proposed a novel computational method based on deep learning to identify DTIs.

Firstly, GSRF-DTI constructed the drug and target homogeneous networks based on multiple drug and target association information and the DTP-NET based on known drug and target sets. Then, Deepwalk was used to learn drug features and target features on the homogeneous networks, with the initial feature of DTPs obtained by concatenating corresponding drug and target features. Next, GraphSAGE is utilized to learn potential network features on DTP-NET. Finally, RF was applied to predict the DTIs. Through comparison with five other state-of-the-art DTI prediction algorithms using Luo’s dataset and the newly constructed dataset, GSRF-DTI demonstrated superior performance.

In addition, we evaluated the influence of the aggregation function, learning_rate, and classification algorithm on the performance of GSRF-DTI. More importantly, we conducted multiple sets of ablation experiments to demonstrate the necessity of integrating various types of network information and the effectiveness of GraphSAGE. Furthermore, we validated the significance of considering interactions between drug-target pairs (DTPs) for DTI prediction. Finally, we utilized GSRF-DTI to identify potential DTIs and verified its effectiveness through case studies.

Previous researchers have predominantly concentrated on the impact of multiclass heterogeneous information on DTIs or the association information between DTPs. In GSRF-DTI, we extensively considered the effects of both these aspects. Furthermore, we applied GraphSAGE, an inductive representation learning method on large graphs, to DTP-NET, resulting in a significant enhancement of prediction performance for DTIs.

In future research, it will be important to concentrate on diverse embedding representations and dimensionality reduction techniques to preserve comprehensive feature information effectively while also devising novel strategies to address the issue of data imbalance.

## Conclusion

In this paper, we proposed a hybrid approach based on network and deep learning named GSRF-DTI to identify DTIs. We tested our model on Luo et al. dataset as well as on a newly constructed dataset, and the experimental results showed that the GSRF-DTI framework outperformed several state-of-the-art methods significantly. Additionally, by comparing results from multiple experiments, we concluded that integrating various types of network information is crucial for identifying drug-target interactions, with protein sequences and disease information proving particularly important. Finally, we applied GSRF-DTI to predict drug-target interactions and proposed three novel DTIs and through the literature search to authenticate them.

Advancements in innovative combinatorial methods for predicting drug-target interactions could facilitate the broader application of these tools in addressing related biological challenges, such as the interaction between micro-RNA and diseases [46, 47] and the association between micro-RNA molecules [48].

### Supplementary Information

Additional file 1. Containing the datasets used in this paper.

Additional file 2: S1-S4. S1-[Datasets] Details of the datasets including Luo’s dataset and the newly constructed dataset. S2-[ Experimental Settings]: The details of experimental settings including the introduction of baseline methods and parameter settings. S3-[GSRF-DTI Hyper-Parameter Optimization]: The details of GSRF-DTI hyperparameter optimization including optimization for learning_rate and aggregation functions. S4-[ GSRF-DTI Model Optimization]: The details of GSRF-DTI Model Optimization.

## Data Availability

All data generated or analyzed during this study are included in this published article, its supplementary information files, and publicly available repositories. In addition, the data and code used during the current study is available at 10.5281/zenodo.12589490.

## Abbreviations

DTI: Drug-target interaction
DTP: Drug-target pair
DTP-NET: Drug-target pair network
RF: Random forest
GraphSAGE: Graph Sample and Aggregate
DDI: Drug-drug interaction
TTI: Target-target interaction
AUROC: The area under the receiver operating characteristics curve
AUPR: The area under the precision and recall curve
LR: Logistic regression
SVM: Support vector machine
