# Supplementary material for: GSRF-DTI: a framework for drug-target interaction prediction based on a drug-target pair network and representation learning on a large graph
Source: BMC Biol. 2024 Jul 18;22:156. doi: 10.1186/s12915-024-01949-3 (PMC11256582; doi:10.1186/s12915-024-01949-3)
Supplement: Supplementary file 2 — Additional file 2: S1-S4. S1-[Datasets] Details of the datasets including Luo’s dataset and the newly constructed dataset. S2-[ Experimental Settings]: The details of experimental settings including the introduction of baseline methods and parameter settings. S3-[GSRF-DTI Hyper-Parameter Optimization]: The details of GSRF-DTI hyperparameter optimization including optimization for learning_rate and aggregation functions. S4-[ GSRF-DTI Model Optimization]: The details of GSRF-DTI Model Optimization. [file 12915_2024_1949_MOESM2_ESM.docx]

**GSRF-DTI:** **Drug-target interaction prediction framework based on drug-target pairs network and**

**representation learning on large graph**

Additional file

# S1 Datasets

To evaluate the performance of a drug-target interaction prediction algorithm based on a drug-target bipartite network and graph representation learning, we tested our model on Luo et al. dataset as well as on the newly constructed dataset.

**S1.1 The dataset of Luo et al.**

The number of various biological entities and the interactions among them are shown in **Table S1** and **Table S2**, respectively.

**Table S1.** Information on the entities in Luo’s dataset

| **Type of node** | **Number** | **Database** | **Address** |
| --- | --- | --- | --- |
| Drug | 708 | DrugBank | http://go.drugbank.com/ |
| Protein | 1512 | HPRD | http://www.hprd.org/ |
| Disease | 5603 | Comparative Toxicogenomics | http://ctdbase.com/ |
| Side effect | 4192 | SIDER | http://sideeffects.embl.de/ |

**Table S2.** Information on the relationships between the entities in Luo’s dataset

| **Interactions between nodes** | **Number** | **Database** | **Address** |
| --- | --- | --- | --- |
| Drug-Protein | 1923 | DrugBank | http://go.drugbank.com/ |
| Drug-Drug | 10036 | DrugBank | http://go.drugbank.com/ |
| Drug-Disease | 199214 | Comparative Toxicogenomics | http://ctdbase.com/ |
| Protein-Disease | 1596745 | Comparative Toxicogenomics | http://ctdbase.com/ |
| Drug-Side-effect | 80164 | SIDER | http://sideeffects.embl.de/ |
| Protein-Protein | 7363 | HPRD | http://www.hprd.org/ |

**S1.2 The newly constructed dataset**

The number of various biological entities and the interactions among them are shown in **Table S3**.

**Table S3.** Information on the relationships between the entities in the newly constructed dataset

| **Interactions between nodes** | **Number** | **Database** | **Address** |
| --- | --- | --- | --- |
| Drug-Protein | 481 | KEGG,  BRENDA,  SuperTarget,  DrugBank | <http://www.genome.jp/kegg/>  http://www.brenda. uni‐koeln.de  <http://insilico.charite.de/supertarget>  <http://go.drugbank.com/> |
| Drug-Drug | 514 | KEGG | http://go.drugbank.com/ |
| Drug-Disease | 57179 | Comparative Toxicogenomics | http://ctdbase.com/ |
| Protein-Disease | 327854 | Comparative Toxicogenomics | http://ctdbase.com/ |
| Drug-Side-effect | 19603 | SIDER | http://sideeffects.embl.de/ |
| Protein-Protein | 628 | KEGG | http://www.hprd.org/ |

# S2 Experimental Settings

**S2.1 The introduction of baseline methods**

In this paper, we compare five algorithms. They are BLMNII, NRLMF, DTI-NET, GCN-DTI and EEG-DTI.

**·BLMNII** is a model that integrates the neighbor-based interaction profile inference method into the bipartite local model approach for predicting drug-target interactions. By using the inferred interaction profiles as labels for biological entities, this method has good performance in predicting targets for new drugs as well as identifying drugs for new targets.

**·NRLMF** uses matrix decomposition to calculate the probability of drug and target interaction based on the specific potential vector representation of the drug and target. To improve the effectiveness of DTI prediction, the method takes the influence of the local structure of the drug and target into consideration, and regularization constraints are applied to the drugs, targets and their nearest neighbors.

**·DTI-NET** is a prediction model of drug-target interactions based on heterogeneous information. In this method, drug similarity matrices and protein similarity matrices are calculated by integrating multiple drug-related information and protein-related information. Based on the above similarity matrices, the low-dimensional feature representation of drugs and proteins is obtained by compact feature learning. Finally, a projection matrix is used to predict DTIs.

**·GCN-DTI** is a network-based drug-target interaction prediction framework that combines convolutional neural networks and deep neural networks. In this model, drug-drug interactions, protein-protein interactions and drug-protein interactions are considered to construct drug-target pair networks, and the problem of predicting connections between nodes is transformed into a node binary classification problem. Based on the drug-target pair network, the graph convolutional neural network is first used to learn the features of the nodes in the network, and then the deep neural network is used to classify the nodes based on the learned features to achieve the prediction of DTIs.

**·EEG-DTI** is a DTI prediction framework for end-to-end heterogeneous graph representation learning. The method constructs a heterogeneous network by integrating the four biological entities of drugs, proteins, diseases and side effects and their interactions. Based on this network, a graph convolution neural network model is used to learn the low-dimensional feature representation of nodes in the network for predicting DTIs.

**S2.2 Parameter Settings**

The performance of the graph neural network model was closely related to the setting of the parameters. In the GSRF-DTI model, we used the Deepwalk algorithm and GraphSAGE algorithm to aggregate neighbor information and learn the potential network features. The following describes the parameter settings for the two algorithms.

In the Deepwalk algorithm, we used the default parameters $\omega=5,\gamma=80$and $t=10$ to learn the drug features $F_{D}$ and target features $F_{T}$, where$\omega$,$\gamma$,$t$ denote window size, walks per size and walk length, respectively. Based on the previous experience, the output dimension of the Deepwalk algorithm was set to 100, that is, a 100-dimensional drug/target feature representation was learned. Therefore, the initial features of DTPs in DTPs-NET are 200-dimensional.

In the GraphSAGE algorithm, we sampled 2-layer neighbors of each node, where 50 neighbor nodes were sampled in the first layer and 10 in the second layer. In this 2-layer neural network, the parameters were constantly adjusted in light of the evaluation metric values.

We let the number of network neurons in the first layer be 64 and the second be 32. Then, F.relu was chosen as the activation function, and mean aggregator was chosen as the aggregation function. Considering the time complexity and the performance of the model, we set batch_sizes, epoch and learning_rate to 300, 50 and 0.001, respectively. In addition, we let dropout equal 0.2 to prevent overfitting of the model during training.

# S3 GSRF-DTI Hyper-Parameter Optimization

**S3.1 Hyper-Parameter : Learning_rate**

**Figure S1** depicts the AUROC performance of the five-fold cross validation with$epoch= 10, 30, 50, 80, 100$ for different learning_rate.


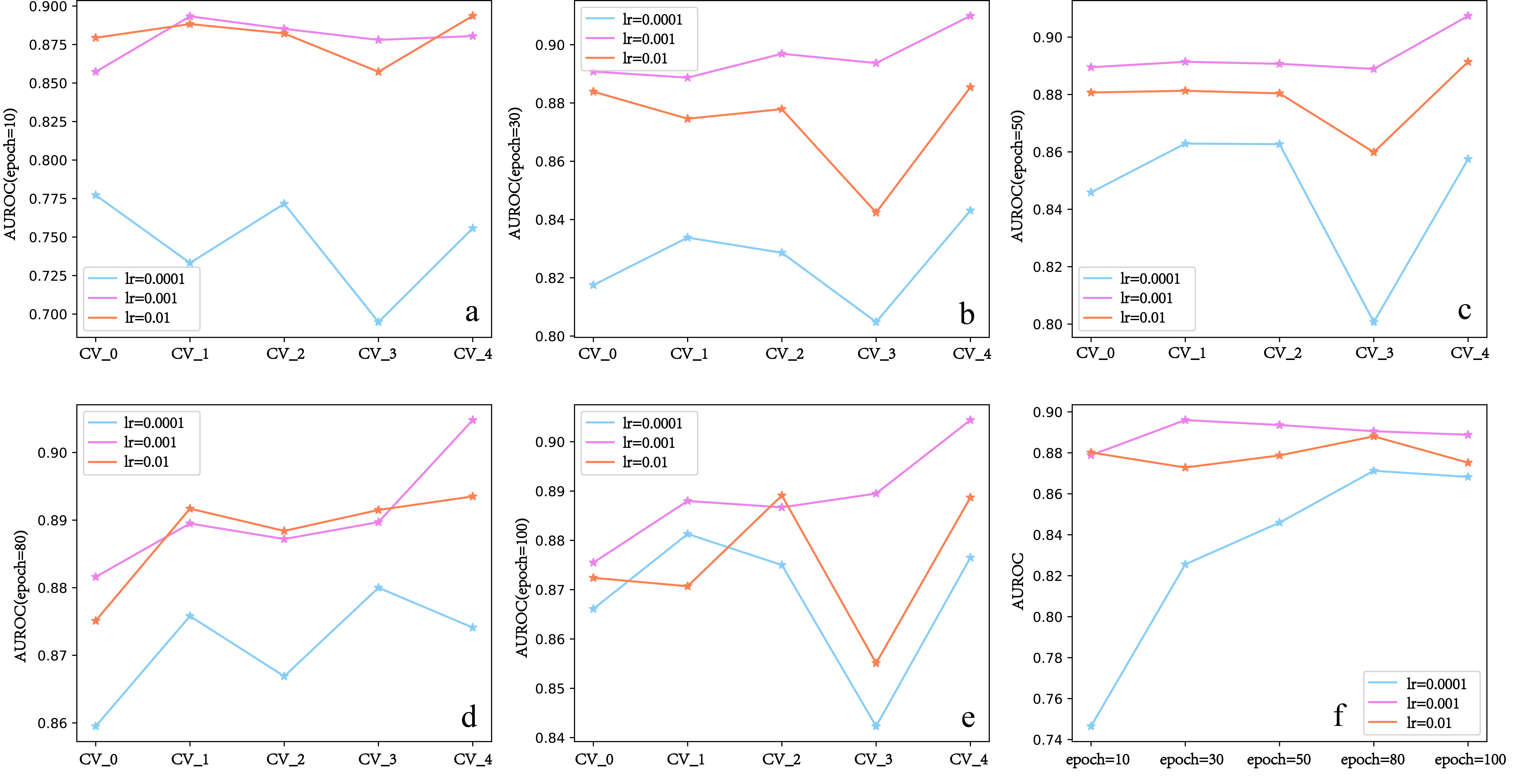


**Figure S1.** The three broken lines in (a) correspond to the AUROC specific results of the five-fold cross validation with the learning_rate equal to 0.01, 0.001 and 0.0001 when$epoch =10$. Similarly, (b), (c), (d) and (e) show the AUROC specific results of five-fold cross validation with learning_rate equal to 0.01, 0.001 and 0.0001 when $epoch= 30, 50, 80, 100$ respectively. The three broken lines in (f) correspond to the average results at learning rates of 0.01, 0.001 and 0.0001 for different epochs.

**Figure S2** depicts the AUPR performance of the five-fold cross validation with$epoch= 10, 30, 50, 80, 100$ for different learning_rate.


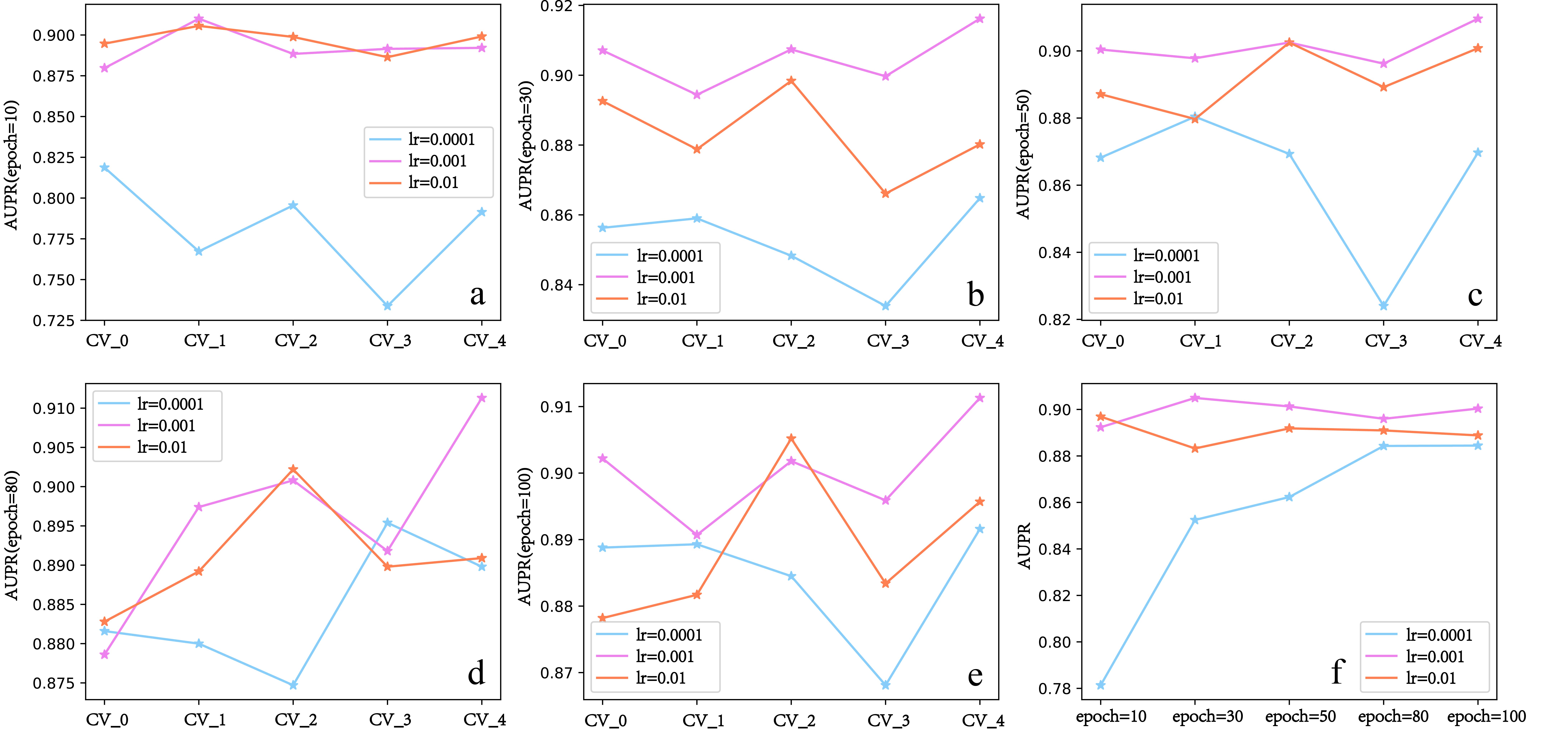


**Figure S2.** The three broken lines in (a) correspond to the AUPR specific results of the five-fold cross validation with the learning_rate equal to 0.01, 0.001 and 0.0001 when$epoch =10$. Similarly, (b), (c), (d) and (e) show the AUPR specific results of five-fold cross validation with learning_rate equal to 0.01, 0.001 and 0.0001 when $epoch= 30, 50, 80, 100$ respectively. The three broken lines in (f) correspond to the average results at learning rates of 0.01, 0.001 and 0.0001 for different epochs.

**S3.2 Hyper-Parameter: Aggregation functions**

To assess the impact of different aggregation functions on model performance, we calculate evaluation index of model prediction results under each aggregation function, the average AUROC and AUPR results of fivefold cross-validation are shown in **Figure S3** Notice that we set learning_rate to 0.001 at this point.

**Figure S3** depicts the AUROC performance of the five-fold cross validation with$epoch= 10, 30, 50, 80, 100$ for different aggregation functions.


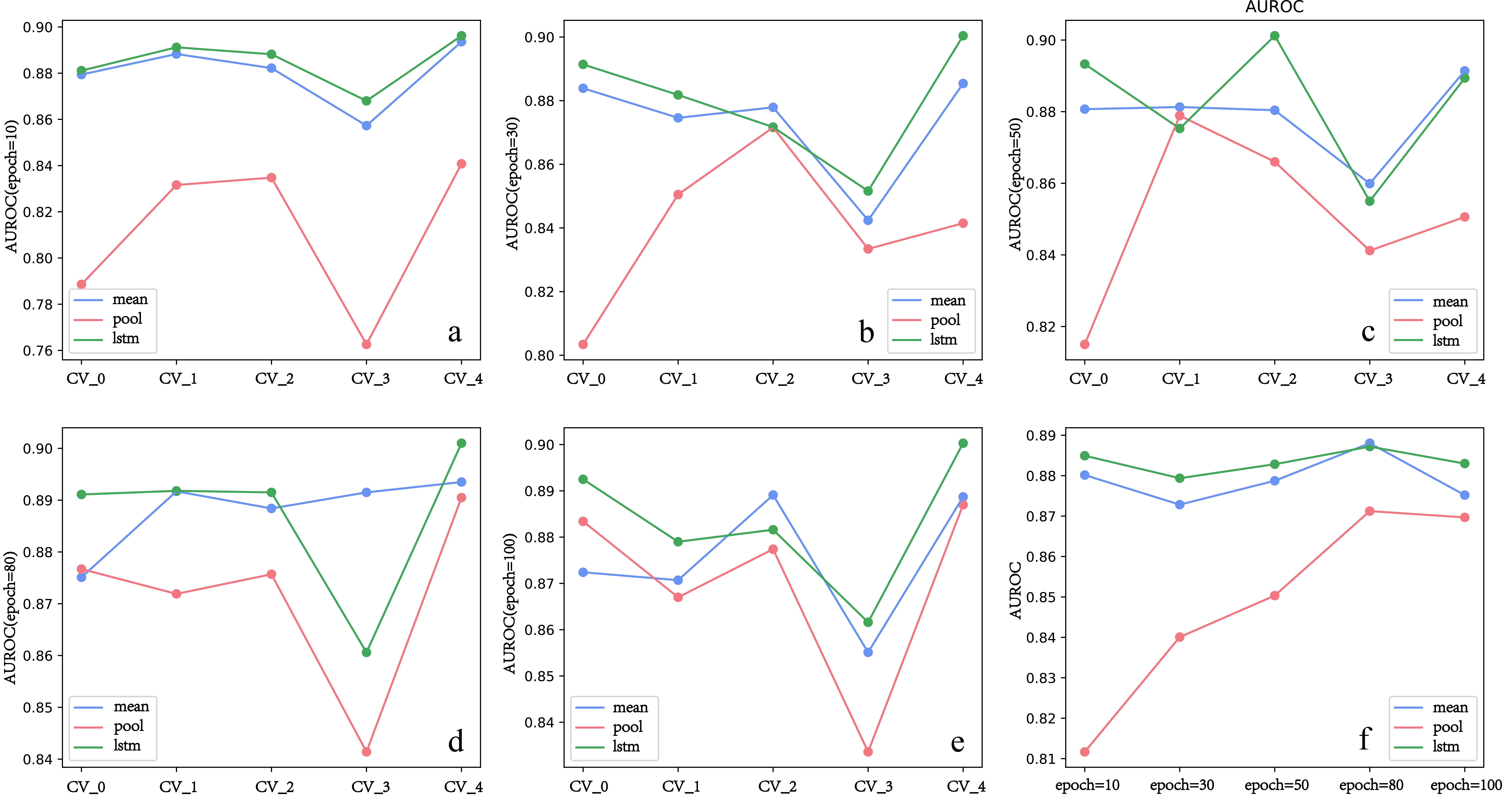


**Figure S3.** The three broken lines in (a) correspond to the AUROC specific results of the five-fold cross validation with the aggregation function are Mean aggregator, Pooling aggregate and LSTM aggregator when$epoch =10$. Similarly, (b), (c), (d) and (e) show the AUROC specific results of five-fold cross validation with aggregation function are Mean aggregator, Pooling aggregate and LSTM aggregator when $epoch= 30, 50, 80, 100$ respectively. The three broken lines in (f) correspond to the average results at aggregation function are Mean aggregator, Pooling aggregate and LSTM aggregator for different epochs.

**Figure S4** depicts the AUPR performance of the five-fold cross validation with$epoch= 10, 30, 50, 80, 100$ for different aggregation functions.


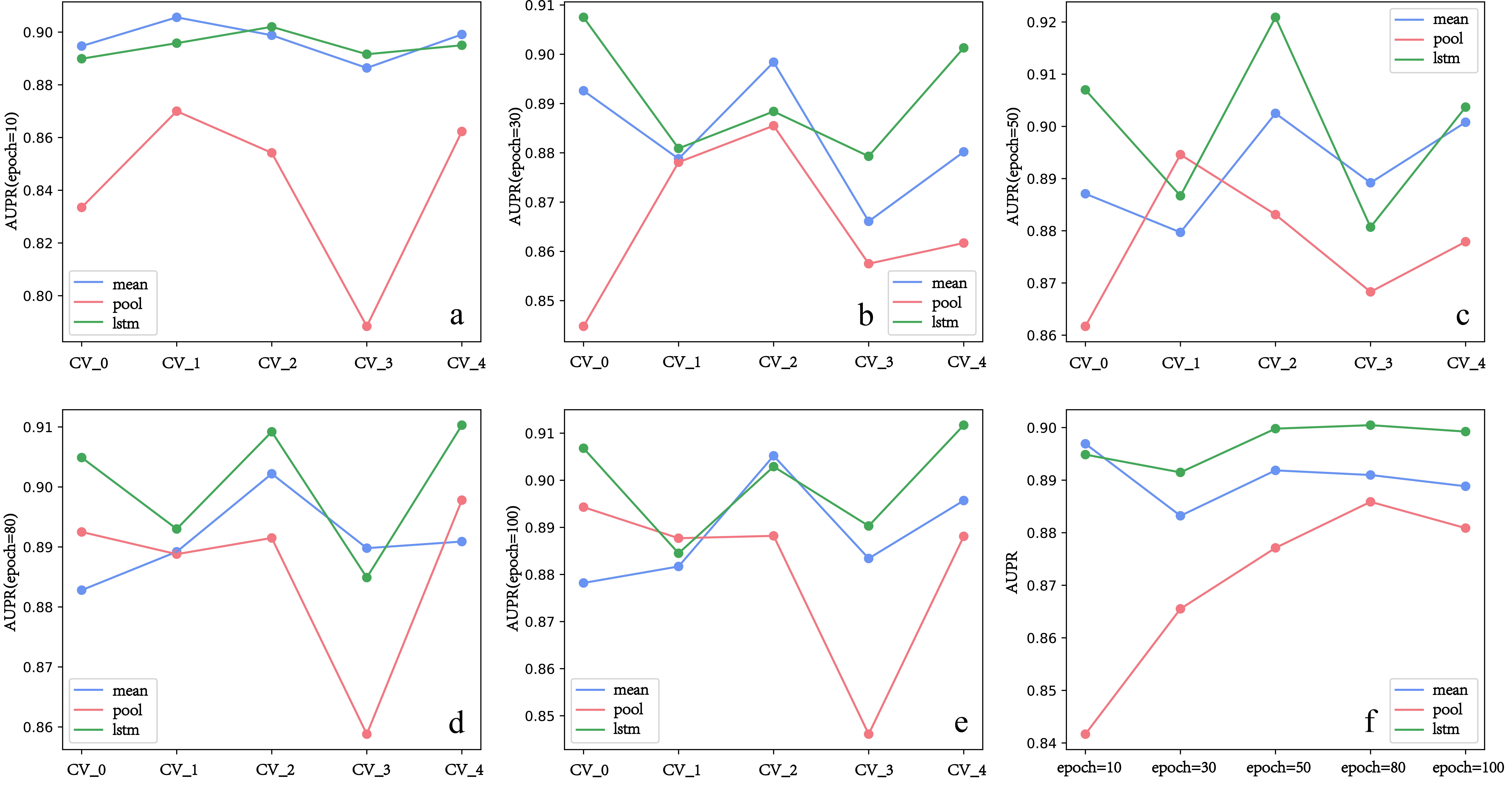


**Figure S4.** The three broken lines in (a) correspond to the AUPR specific results of the five-fold cross validation with the aggregation function are Mean aggregator, Pooling aggregate and LSTM aggregator when$epoch =10$. Similarly, (b), (c), (d) and (e) show the AUPR specific results of five-fold cross validation with aggregation function are Mean aggregator, Pooling aggregate and LSTM aggregator when $epoch= 30, 50, 80, 100$ respectively. The three broken lines in (f) correspond to the average results at aggregation function are Mean aggregator, Pooling aggregate and LSTM aggregator for different epochs.

# S4 GSRF-DTI Model Optimization

To illustrate that associations between DTPs have an important impact on DTI prediction, GSRF-DTI designs contrast experiments. In the basic experiment, the initial features of the obtained DTPs are directly used as the input of the three classical binary classification algorithms (LR, SVM and RF), and 50 random experiments are performed, respectively. In the contrast experiment, the features of DTPs based on the action of GraphSAGE algorithm are used as the input of the three classical binary classification algorithms, and 50 random experiments are performed, respectively.

**Table S4** depicts the performance of the six metrics under the five-fold cross validation for the different classifiers.

**Table S4.** The performance of the different classifiers.

| **Method** | **Val** | **Acc** | **Precision** | **Reacll** | **F1** | **AUROC** | **AUPR** |
| --- | --- | --- | --- | --- | --- | --- | --- |
| G_SVM | val0 | 0.9611 | 0.9629 | 0.9601 | 0.9614 | 0.9815 | 0.9823 |
| G_SVM | val1 | 0.9572 | 0.9636 | 0.9508 | 0.9571 | 0.9794 | 0.9803 |
| G_SVM | val2 | 0.9563 | 0.9634 | 0.9495 | 0.9563 | 0.9807 | 0.9796 |
| G_SVM | val3 | 0.9554 | 0.966 | 0.9448 | 0.9552 | 0.9772 | 0.9816 |
| G_SVM | val4 | 0.9606 | 0.9612 | 0.9607 | 0.9609 | 0.9811 | 0.9806 |
| **MEAN** | **VAL** | **0.95812** | **0.96342** | **0.95318** | **0.95818** | **0.97998** | **0.98088** |
| G_LG | val0 | 0.9592 | 0.9593 | 0.9599 | 0.9596 | 0.9803 | 0.9787 |
| G_LG | val1 | 0.9556 | 0.9564 | 0.9554 | 0.9559 | 0.9813 | 0.981 |
| G_LG | val2 | 0.9479 | 0.9491 | 0.9474 | 0.9482 | 0.9815 | 0.9834 |
| G_LG | val3 | 0.9505 | 0.9537 | 0.9477 | 0.9506 | 0.9755 | 0.9799 |
| G_LG | val4 | 0.9594 | 0.9591 | 0.9605 | 0.9598 | 0.9802 | 0.9811 |
| **MEAN** | **VAL** | **0.95452** | **0.95552** | **0.95418** | **0.95482** | **0.97976** | **0.98082** |
| G_RF | val0 | 0.9609 | 0.9646 | 0.9577 | 0.961 | 0.9826 | 0.9855 |
| G_RF | val1 | 0.9569 | 0.9621 | 0.952 | 0.9569 | 0.9827 | 0.985 |
| G_RF | val2 | 0.9564 | 0.962 | 0.951 | 0.9564 | 0.9823 | 0.983 |
| G_RF | val3 | 0.9558 | 0.9647 | 0.947 | 0.9557 | 0.978 | 0.9821 |
| G_RF | val4 | 0.959 | 0.9597 | 0.959 | 0.9593 | 0.9833 | 0.9839 |
| **MEAN** | **VAL** | **0.9578** | **0.96262** | **0.95334** | **0.95786** | **0.98178** | **0.9839** |

In the **Table S4**, G_SVM, G_LG, G_RF denote the simultaneous use of GraphSAGE and SVM algorithms, GraphSAGE and Logistic Regression algorithms, and GraphSAGE and Random Forest algorithms, respectively. $\text{Val} i ,i=0,1,\cdots,4$ represents the $i+1\text{-th}$ fold in the five-fold cross validation.
